# Meta-analysis of factors affecting milk component yields in dairy cattle

**DOI:** 10.1186/2055-0391-56-5

**Published:** 2014-06-05

**Authors:** Junsung Lee, Jakyeom Seo, Se Young Lee, Kwang Seok Ki, Seongwon Seo

**Affiliations:** Department of Animal Biosystem Sciences, College of Agriculture and Life Science, Chungnam National University, Daejeon, 305-764 Republic of Korea; Department of Animal Husbandry, Cheonan Yonam College, Cheonan, 330-802 Korea; National Institute of Animal Science, RDA, Cheonan, 330-801 Korea

**Keywords:** Meta analysis, Milk fat, Milk protein, Dry matter intake, Dairy cattle

## Abstract

The objectives of this study were thus to identify most significant factors that determine milk component yield (MCY) using a meta-analysis and, if possible, to develop equations to predict MCY using variables that can be easily measured in the field. A literature database was constructed based on the research articles published in the *Journal of Dairy Science* from Oct., 2007 till May, 2010. The database consisted of a total of 442 observed means for MCY from 118 studies. The candidate factors that determine MCY were those which can be routinely measured in the field (e.g. DMI, BW, dietary forage content, chemical composition of diets). Using a simple linear regression, the best equations for predicting milk fat yield(MFY) and milk protein yield (MPY) were MFY = 0.351 (±0.068) + 0.038 (±0.003) DMI (R^2^ = 0.27), and MPY = 0.552 (±0.071) + 0.031 (±0.002) DMI - 0.004 (±0.001) FpDM (%, forage as a percentage of dietary DM) (R^2^ = 0.38), respectively. The best equation for predicting milk fat content (%) explained only 12% of variations in milk fat content, and none of a single variable can explain more than 5% of variations in milk protein content. We concluded that among the tested variables, DMI was the only significant factor that affects MFY and both DMI and FpDM significantly affect MPY. However, predictability of linear equations was relatively low. Further studies are needed to identify other variables that can predict milk component yield more accurately.

## Background

The manipulation of milk composition has been of interest to improve the nutritional value of milk and to increase the efficiency in manufacturing and processing of raw milk for dairy products (Jenkins and McGuire, [1]). In addition, the decision of milk price depends on the amounts of milk component yield (MCY) in most countries including Korea, performing of quantitative analysis of factors that influence on MCY is crucially important in dairy industry.

Many studies have tried to identify such factors affecting milk yield (MY) and MCY for past decades. DMI, dietary energy and protein or individual carbohydrate (CHO) and protein fractions might be important factors in controlling MY and milk protein yield (MPY) in dairy cows ((Sutton, [2], Grummer, [3], DePeters and Cant, [4], Palmquist et al., [5], Hristov et al., [6, 7], Jenkins and McGuire, [1], Huhtanen and Hristov, [8]). Smoler et al. [9] suggested individual CHO fractions might be better predictors of MPY than total CHO. Hristov et al. [10] showed a moderate linear relationship (R^2^ = 0.47) between DMI and MY using data set published in *Journal of Dairy Science* (Volumes 1 through 82). According to NRC [11], dietary CP was not correlated (P > 0.25) with milk protein percent, but was correlated weakly (r = 0.14; P < 0.01) with MPY. Huhtanen and Hristov [8] reported that metabolizable protein (MP) intake was better predictor of MPY compared with CP intake.

In addition, there have been attempts to develop equations to predict MY and MPY of dairy cows (NRC, [11], Hristov et al., [6]) through a meta-analysis approach. They, however, included RDP and RUP in the equation, which cannot be easily measured in the field. NRC [11] also presented equations to predict MY and MPY with DMI and CP contents in diets; however, the predictability was insufficient and study effect was not accounted for in developing equations. The objectives of this study were thus to identify most significant factors that determine MCY using meta-analysis based on recent studies conducted from last decade and, if possible, to derive equations to predict MCY with variables that can be easily measured in the field.

## Results and discussions

Animal parameters, nutrient composition of diet, milk yield and composition were listed in Table 1. The variations in each variables used for developing equation in this study was large enough to represent a wide range of data.

**Table 1:** **Descriptive statistics for the database used for developing equations in this study**

	N	Mean	S.D.	Median	Max	Min
Animal inputs						
BW, kg	442	638.21	53.41	641.00	778.00	443.00
DIM, day	358	115.23	56.12	116.50	277.00	1.00
DMI, kg/d	442	22.53	3.51	22.95	32.80	7.00
DMI,% BW	442	3.53	0.50	3.60	5.04	1.26
Forage DMI, kg	442	11.40	2.49	11.40	19.90	3.47
Forage,%DM	442	51.06	10.66	50.00	100.00	19.70
Nutrient composition						
DM,% AF	303	54.90	9.58	53.90	88.40	27.73
CP,% DM	441	17.35	1.89	17.30	25.98	7.10
EE,% DM	203	4.35	1.30	4.35	8.40	1.50
Ash,% DM	189	7.80	2.08	7.50	18.40	3.42
NFC,% DM	183	38.52	5.72	39.30	51.40	24.60
NDF,% DM	438	33.19	4.81	32.70	51.80	19.50
ADF,% DM	340	20.73	3.89	20.20	37.40	11.20
RDP,% DM	116	10.64	1.17	10.60	13.17	6.89
RUP,% DM	103	7.36	5.20	6.30	33.70	4.40
Starch,% DM	174	23.82	5.77	23.30	39.20	9.50
NEL, Mcal/kg	249	1.62	0.08	1.61	1.80	1.40
Milk yield and composition						
Milk yield, kg/d	442	34.53	6.76	35.55	50.90	15.70
3.5% FCM, kg	442	34.68	6.74	35.45	52.11	16.18
4.0% FCM, kg	442	32.08	6.23	32.79	48.20	14.97
Fat,%	442	3.57	0.51	3.53	5.93	2.16
Fat yield, kg	442	1.22	0.26	1.24	1.96	0.55
Protein,%	442	3.14	0.37	3.09	6.87	2.53
Protein yield, kg	442	1.06	0.22	1.09	1.73	0.47
MUN, mg/dl	242	13.63	4.34	13.01	35.80	6.77

The best equation for predicting milk fat content (%) explained only 12% of the variations in milk fat content, and none of a single variable could explain more than 5% of the variations in milk protein content. According to the review by Jenkins and McGuire [1], the most sensitive component of milk to dietary manipulation was fat content, which could be changed over a range of 3 percentage units. Milk protein was more responsive to diet (over a 0.5 percentage unit range) than lactose, but less responsive than fat. Sutton [2] reported that milk fat concentration was affected by the amount of roughage, the forage-to-concentrate ratio, the carbohydrate composition of concentrate mix, lipids, intake, and meal frequency. A reduction in the dietary forage-to-concentrate ratio usually decreases milk fat content although the degree of response varies (Sutton, [2]). Milk fat content was fairly stable until the proportion of forage in the diet on a DM basis falls to about 50%, but with further reductions in the proportion of forage, a decrease in milk fat content occurs (Thomas and Martin, [12]). Smith et al. [13] indicated that the response in milk fat content to dietary supplementation of lipid was highly variable by the amount, physical form, and fatty acid composition of lipid. Sporndly [14] observed no significant correlation between protein content of milk and protein concentration of the diet (r = 0.06), while milk protein yield and dietary protein level were correlated (r = 0.37). Jenkins and McGuire [1] reported that reducing the proportion of forage in the diet increased both protein content and yield. Milk protein content could increase by 0.4 percentage units or more when forage proportion in the diet reduced to 10% or less of the diet DM. In addition, they indicated that low transfer efficiency (25 to 30%) of dietary protein to milk was a major factor accounting for the inability of diet to markedly alter milk protein content. Therefore, large variations or low responses to dietary manipulation resulted in low predictability for milk fat and protein content with dietary factors.

**Figure 1:**
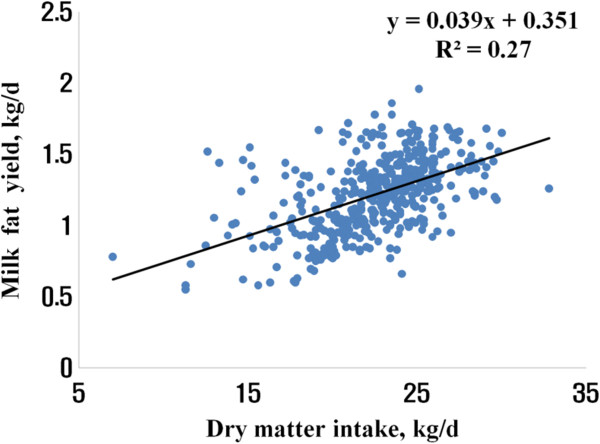
**Relationship of milk fat yield with dry matter intake.**

**Figure 2:**
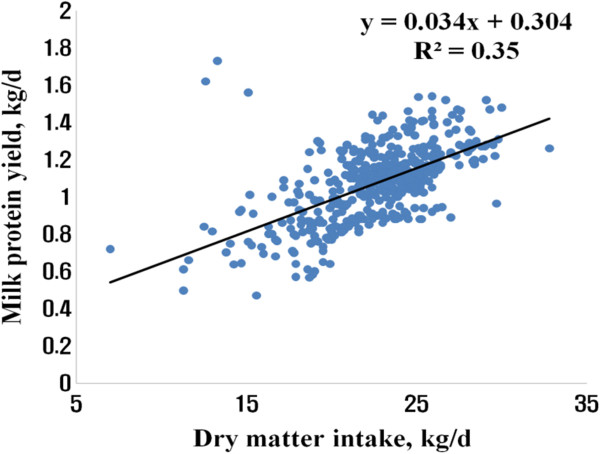
**Relationship of milk protein yield with dry matter intake.**

**Figure 3:**
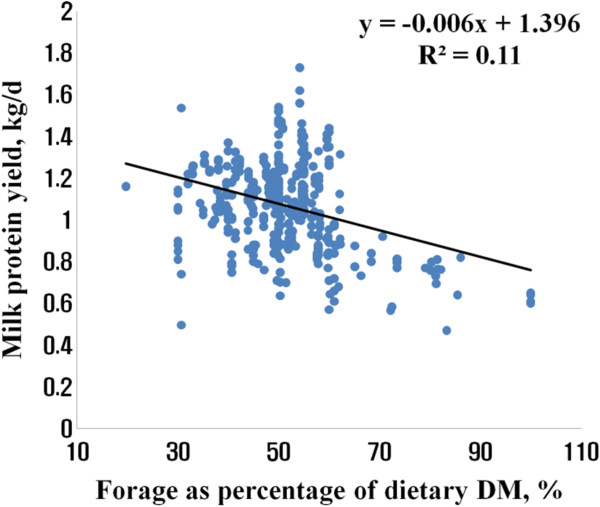
**Relationship of milk protein yield with forage as percentage of dietary DM.**

A further analysis was done to develop equations with variables that can be easily measured in the field for predicting MPY and MFY. DMI alone explained 27% of variations in MFY (Figure 1) and 35% of variations in MPY (Figure 2). There was negative correlation between MPY and FpDM (Figure 3), which was consistent with other report (DePeters and Cant, [4]). DePeters and Cant [4] indicated that the negative effect of forage content in the diet on MPY was related with reduction in energy density of the diet. Positive correlations between both the amount and the concentration of metabolizable energy and either milk protein content or protein yield were observed (Sporndly, [14]). However, an increase in energy intake by adding supplementary fat in the diet normally resulted in reduced milk protein content (Emery, [15]).

Using a random coefficient model with study as a random effect, we obtained 0.311 (±0.072) + 0.041 (±0.003) DMI (n = 442, -2 Res log likelihood = - 499) and 0.497 (±0.084) + 0.032 (±0.002) DMI - 0.003 (±0.001) FpDM (n = 442, -2 Res log likelihood = -721) for predicting MFY and MPY, respectively (Table 2). Unlike the model presented in NRC [11], CP content in the diet or CP intake was not significant variable to predict MPY. Using a simple linear regression, the best equations for predicting MFY and MPY were MFY = 0.351 (±0.068) + 0.038 (±0.003) DMI (R^2^ = 0.27, root mean square error (RMSE) = 0.22), and MPY = 0.552 (±0.071) + 0.031 (±0.002) DMI - 0.004 (±0.001) FpDM (R^2^ = 0.38, RMSE = 0.16), respectively.

**Table 2:** **Estimates, standard errors and significance for the random components of the final candidate models for milk component yield (kg/d; n = 442)**

Item ^*^	Parameter	Estimate	SE	P > |z|
MFY	Intercept	0.311	0.072	<0.0001
	DMI	0.041	0.003	0.0014
MPY	Intercept	0.497	0.084	<0.0001
	DMI	0.032	0.002	<0.0001
	FpDM	-0.003	0.001	0.0101

^*^MFY; milk fat yield, MPY; milk protein yield.

The relationship of MCY with diet composition variables and intake of specific nutrients in dairy cows was also reported by previous studies (Sutton, [2], Grummer, [3], DePeters and Cant, [4], Palmquist et al., [5], Hristov et al., [6, 7], Jenkins and McGuire, [1], Huhtanen and Hristov, [8]).The supplementation of lipids at up to 6 to 8% in the diet DM generally increases milk yields but the response in milk fat content varies widely, so that milk fat yield usually remains unchanged or increases (Macleod and Wood, [16], Van der Honing et al., [17]). Rook et al. [18] reported that DMI, dietary NDF concentration, and digestibility of dietary OM were important in predicting MPY. Sporndly [14] observed crude protein intake was correlated positively with milk protein content (r =0.25) and protein yield (r = 0.69). In addition to dietary CP, estimated MP (NRC, [11]), intakes of RDP or RUP were significant predictors for MPY in dairy cows (Smoler et al., [9], NRC, [11], Hristov et al., [7]). Hristov et al. [7] showed the negative correlation of MPY with fermentable NDF and protein fraction B1 intakes. Diets for lactating dairy cows were formulated to be highly digestible and, in most situations, DMI was strongly related to intake of total digestible nutrients or energy (net energy for lactation or metabolizable energy) intake (Hristov et al., [19]). In Huhtanen and Hristov [8] the best prediction models for MPY were based on total digestible nutrients (TDN), CP intake and CP degradability. Hristov et al. [6] also suggested MY and MPY in dairy cows can be better predicted based on intake of individual nutrients than DMI alone.

## Conclusions

Because genetic traits as well as production performance of dairy cows have been improved, a new development of equations for predicting MCY on the basis of animal parameters and/or feed characteristics was needed. Therefore, the recent experimental data obtained from 10 years before was used to re-evaluate major factors affecting MCY through meta-analysis in this study. We concluded that MFY and MPY can be predicted by DMI and FpDM which can easily be measured in field. However, predictability was relatively low. There was no variable or a combination of variables that were routinely measured and can be used to predict milk composition. More researches are needed to identify other variables that can predict milk component yield and milk composition more accurately.

## Methods

### Database construction

The database consisted of a total of 442 treatment means from 118 studies published in the *Journal of Dairy Science* from Oct., 2007 till May, 2010 (volumes 90 through 93). The detailed descriptive statistics of the database was described in Table 1. The average (±SD) body weight (BW) of the cows involved in this study was 638.2 ± 53.41 kg with a minimum of 443 kg and a maximum of 778 kg. The average DMI were 22.53 ± 3.51 kg/d with a minimum of 7.0 and a maximum of 32.8 kg/d. In nutrient composition of diets, average DM, CP, EE, NDF, RDP and RUP were 54.9 ± 9.58%, 17.4 ± 1.89%, 4.4 ± 1.30%, 33.2 ± 4.81%, 10.6 ± 1.17% and 7.4 ± 5.20%, respectively. Average MY was 34.53 ± 6.76 kg/d with 3.57 ± 0.51% of average milk fat content and 3.14 ± 0.37% of average milk protein content. Average MFY and MPY were 1.22 ± 0.26 and 1.06 ± 0.22 kg, respectively.

### Model development and statistical analyses

The independent variables used for explaining the variations in MCY (i.e. MPY, MFY, milk protein content in milk, and milk fat content in milk) were DMI (kg/d), BW (kg), DMIpBW (%, dry matter intake as a percentage of BW), FpDM (%, forage as a percentage of dietary DM), NDF (% DM), CP (% DM), starch (% DM), ADF (% DM), CP intake (kg/d), NDF intake (kg/d), starch intake (kg/d), and forage intake (kg/d). Among these variables, four to five predictive variables that sufficiently explained the variations in each dependent variable were selected using step-wise regression.

The regression equations were developed in two phases. In the first phase, a random coefficients model was used using the MIXED procedure of SAS (SAS Institute, Inc., Cary, U.S.A) with studies as a random variable to identify independent variables that were statistically significant for each equation.


where y is the vector of observed MCY of which size is N; X the n × p matrix of x_i,j_; β the fixed effects parameter vector of which size is p; Z the designed N × (s × p) matrix that was blocked diagonally corresponding to each study (n_i_ × p) to account for the random effect of each study; e the unknown vector of independent, identically and normally distributed random errors with mean 0 and variance σ^2^; N the total number of observations; s the number of studies; n_i_ the number of observations that ith study; p the number of parameters, which is one (intercept) plus the number of variables used in the equation; and u and e are the normally distributed.

Among the acceptable regression models that had a linear combination of significant fixed effect variables, a model that had the lowest value of -2 restricted log likelihood, Akaike information criterion (AIC), the corrected Akaike information criterion (AICc) and Schwarz’s Bayesian criterion (SBC) was selected. The lowest value of those criteria above indicates a better model considering the number of observations, the number of parameters, and the maximum likelihood estimates. In the second phase, the parameters of the variables in the best model for each dependent variable, identified in the first phase, were estimated by fitting the prediction equations to a multiple regression model using GLM procedure of SAS (SAS Institute, Inc., Cary, U.S.A) [20].

## References

[1] Jenkins TC, McGuire MA (2006). Major advances in nutrition: Impact on milk composition. J Dairy Sci.

[2] Sutton JD (1989). Altering milk-composition by feeding. J Dairy Sci.

[3] Grummer RR (1991). Effect of feed on the composition of milk-fat. J Dairy Sci.

[4] DePeters EJ, Cant JP (1992). Nutritional factors influencing the nitrogen composition of bovine milk: A review. J Dairy Sci.

[5] Palmquist DL, Beaulieu AD, Barbano DM (1993). Feed and animal factors influencing milk-fat composition. J Dairy Sci.

[6] Hristov AN, Price WJ, Shafii B (2004). A meta-analysis examining the relationship among dietary factors, dry matter intake, and milk and milk protein yield in dairy cows. J Dairy Sci.

[7] Hristov AN, Price WJ, Shafii B (2005). A meta-analysis on the relationship between intake of nutrients and body weight with milk volume and milk protein yield in dairy cows. J Dairy Sci.

[8] Huhtanen P, Hristov AN (2009). A meta-analysis of the effects of dietary protein concentration and degradability on milk protein yield and milk N efficiency in dairy cows. J Dairy Sci.

[9] Smoler E, Rook AJ, Sutton JD, Beever DE (1998). Prediction of milk protein concentration from elements of the metabolizable protein system. J Dairy Sci.

[10] Hristov AN, Hristova KA, Price WJ (2000). Relationship between dry matter intake, body weight, and milk yield in dairy cows: A summary of published data. J Dairy Sci.

[11] NRC (2001). Nutrient Requirements of Dairy Cattle.

[12] SAS, Institute Inc (2002). User's guide: Statistics, Version 9th ed.

[13] Thomas PC, Martin PA (1988). The influence of nutrient balance on milk yield and composition. Nutrition and Lactation in the Dairy Cow.

[14] Smith NE, Dunkley WL, Franke AA (1978). Effects of feeding protected tallow to dairy cows in early lactation. J Dairy Sci.

[15] Sporndly E (1989). Effects of diet on milk composition and yield of dairy cows with specical emphasis on milk protein content. Swed J Agric Res.

[16] Emery RS (1978). Feeding for increased milk protein. J Dairy Sci.

[17] Macleod GK, Wood AS (1972). Influence of amount and degree of saturation of dietary fat on yield and quality of milk. J Dairy Sci.

[18] Van der Honing Y, Weiman BJ, Steg A, Van Donselaar B (1981). The effect of fat supplementation of concentrates on digestion and utilization of energy by productive dairy cows. Neth J Agric Sci.

[19] Rook AJ, Sutton JD, France J (1992). Prediction of the yields of milk constituents in dairy-cows offered silage ad-libitum and concentrates at a flat rate. Anim Prod.

[20] Hristov AN, Price WJ, Shafii B (2002). Proceedings of Pacific Northwest Animal Nutrition Conference. An Overview of Dietary Factors Influencing Dry Matter Intake and Milk and Milk Protein Yields In Dairy Cows.

